# The performative pleasure of imprecision: a diachronic study of entrainment in music performance

**DOI:** 10.3389/fnhum.2014.00863

**Published:** 2014-10-29

**Authors:** Andrew Geeves, Doris J. McIlwain, John Sutton

**Affiliations:** ^1^Departments of Psychology and Cognitive Science, Macquarie UniversitySydney, NSW, Australia; ^2^Department of Psychology, Macquarie UniversitySydney, NSW, Australia; ^3^Department of Cognitive Science, Macquarie UniversitySydney, NSW, Australia

**Keywords:** music performance, entrainment, improvisation, qualitative research, diachronic fieldwork

## Abstract

This study focuses in on a moment of live performance in which the entrainment amongst a musical quartet is threatened. Entrainment is asymmetric in so far as there is an ensemble leader who improvises and expands the structure of a last chorus of a piece of music beyond the limits tacitly negotiated during prior rehearsals and performances. Despite the risk of entrainment being disturbed and performance interrupted, the other three musicians in the quartet follow the leading performer and smoothly transition into unprecedented performance territory. We use this moment of live performance to work back through the fieldwork data, building a diachronic study of the development and bases of entrainment in live music performance. We introduce the concept of entrainment and profile previous theory and research relevant to entrainment in music performance. After outlining our methodology, we trace the evolution of the structure of the piece of music from first rehearsal to final performance. Using video clip analysis, interviews and field notes we consider how entrainment shaped and was shaped by the moment of performance in focus. The sense of trust between quartet musicians is established through entrainment processes, is consolidated via smooth adaptation to the threats of disruption. Non-verbal communicative exchanges, via eye contact, gesture, and spatial proximity, sustain entrainment through phase shifts occurring swiftly and on the fly in performance contexts. These exchanges permit smooth adaptation promoting trust. This frees the quartet members to play with the potential disturbance of equilibrium inherent in entrained relationships and to play with this tension in an improvisatory way that enhances audience engagement and the live quality of performance.

## INTRODUCTION

Four professional musicians are performing a particular piece of music together on stage in front of a live paying audience for the eleventh time in their lives. In a moment of improvisation, without any prior discussion with the other three musicians, the member of the quartet who is leading the piece of music extends its structure. Despite being taken by surprise, the other three quartet members are able to adapt to this change. The group members remain synchronized in their music and coordinated in their movement and the performance continues smoothly. Numerous questions arise in relation to this moment in performance: How was the quartet able to continue performing fluidly even though the extension of the length of the piece to this degree had never before been rehearsed or performed? How could the leading member of the quartet trust that the other members of the group would be able to follow his lead without the performance falling into disarray? How could the other quartet members trust that the performance would not fall into disarray when they followed the leading member? What kind of groundwork was laid in the rehearsals and performances preceding this moment that contributed to the three musicians being able to adapt so swiftly to the leading member’s improvisation? Why did the leading member decide to extend the structure of the piece of music on this particular occasion? In what ways, if any, might this moment of improvisation have influenced the audience’s experience of the quartet’s performance? What does this case study add to our understanding of entrainment in naturalistic performance conditions?

Potential answers to some of these questions can be found through an examination of past literature on entrainment and improvisation. Entrainment is found across a range of domains, underlying the synchronisation of pendulum clocks mounted to a common support, firefly illumination, speech rhythms in human conversation, and audience applause (Néda et al., 2000a,b; Clayton et al., 2005). Broadly defined, entrainment is “the process by which independent rhythmical systems interact with each other” (Clayton, 2012, p. 49) in some cases such that “they adjust toward and eventually “lock in” to a common phase and/or periodicity.” (Clayton et al., 2005, p. 2). Rhythmical systems are considered to be independent if the sustenance of their rhythm does not rely on their entrainment to other systems. For such systems to be said to be entrained they must have in common some form of oscillatory activity and be coupled in some way.

Entrainment hinges on the notion of disturbance. As Clayton (2012) emphasises, entrainment does not entail systems falling precisely into phase with each other but, rather, involves the stabilisation of a phase relationship between systems: if we clap together we are entrained with in-phase rhythms, if you consistently clap midway between my claps we are entrained with anti-phase rhythms. Most importantly, for this process to be considered entrainment, the stability of this phase relationship, whether in- or anti-phase, must be re-established after it is disturbed. Entrainment is not always symmetrical. As Clayton et al. (2005) make clear, even within mutual entrainment, a system with greater power or dominance can still exert a disproportionate amount of influence over a relationship. For example, Queen Elizabeth and Prince Philip demonstrate entrainment when walking together on public occasions but the Prince must adapt further in the process of mutual entrainment than the Queen to ensure that he remains three steps behind her at all times. Not only are both the recovery of phase stabilisation after interruption and the potential asymmetry in entrainment important, but these processes can occur implicitly and be inaccessible to awareness. It is unsurprising, therefore, that attention has been found to play a key role in human entrainment (see Jones and Boltz, 1989; Large and Jones, 1999).

Phillips-Silver et al. (2010) suggest that even the simplest forms of entrainment rely upon a rhythmical system possessing three different capacities spanning the ability to: (1) detect rhythmic signals in the environment; (2) produce rhythmic signals; (3) integrate sensory information and motor production so that motor output can be adjusted based on rhythmic input. The example of a dancing couple is provided to illustrate these three building blocks of entrainment. The two dancers are able to: (1) detect the pulse of the song to which they are dancing; (2) produce movement in their feet; (3) integrate their detection of sensory information with their capacity for motor production so that they can move their feet *in time with* the music. The authors also suggest that, in addition to entrainment occurring between the dancers and the external pulse of the music, social entrainment may occur between the dancers so that they adjust their movements based on the rhythmic signals they detect the other producing via auditory, tactile/vestibular or visual cues. Four different types of entrainment are posited: (1) self-entrainment – responding to self-generated rhythmic output; (2) social entrainment – responding to rhythmic output generated by another person; (3) mutual social entrainment – two people responding via a bidirectional information processing loop such that the rhythmic output of person 1 is taken as input by person 2 whose rhythmic output is then taken as input by person 1; (4) collective social entrainment – mutual social entrainment occurring across more than two parties, such that there is a network of input/output connections created between more than two individuals in a group.

Music performance is one domain in which entrainment has been extensively studied. Without referring to Phillips-Silver et al. (2010) and Clayton (2012, 2013) theorises three different manifestations of entrainment in music performance: intra-individual entrainment when a person entrains to his/her own actions, inter-individual/intra-group entrainment when the actions of individuals in a group entrain and inter-group entrainment when different groups entrain. Like entrainment in other domains, entrainment in music performance is considered to be recursive (as it occurs at intra-, inter- and supra-personal levels) and diverse (because it does not necessarily result in in-phase synchronisation of rhythms of matching periods). Leman (2012) argues that, alongside the timing dimension so prominent in Clayton’s account of entrainment, a spatiotemporal dimension rooted in bodily gestures is also needed in order to comprehensively understand the phenomenon in music performance. For Leman, entrainment is unavoidably embodied and he therefore advocates in its study a greater consideration of context, gesture repertoire and sensorimotor cycles.

Empirical research investigating entrainment in music performance has emerged within the last decade. A significant portion of this work has come under the auspices of the “Experience and Meaning in Music Performance” EMMP, 2005–2008) project run out of the Open University and led by Clayton. As Doffman (2011) explains, EMMP researchers have favored a methodology in which fine-grained analysis of audio and visual data gathered from a “real world” music performance given under naturalistic performance conditions is combined with the ethnographic methods of participant observation and interviews. Clayton (2007a,b) drew on this methodology in two studies in which he investigated North Indian *rag* performance. In the first study Clayton (2007a), he investigated the relationship between time, gesture and attention and found that, while eye contact and bodily orientation influenced the dynamics of performance, performing musicians primarily shared the experience of time and motion with each other by making gestures that were either tied to the content of the singing in the performance (named “Illustrators”) or related to music process and structure (named “Markers”). Gesture and eye contact were found to occupy a similarly important position by Moran (2010, 2013) in her analyses of North Indian music performance that also used the EMMP approach to methodology.

In Clayton’s (2007b) second study, one harmonium, one tabla and two tanpura (plucked lute) accompanists backed vocal and tanpura soloist Veena Sahasrabuddhe in another North Indian *rag* music performance. Despite explicitly trying to keep the rhythm of her tanpura separate from that of other instruments and without realising that she was doing so, one tanpura accompanist became entrained with Sahasrabuddhe every time her visual attention was fixed on the soloists’ back. For Clayton, this intra-group entrainment occurring not only outside of the accompanists’ conscious awareness but also in spite of her express efforts to prevent it provides evidence for the strength of entrainment processes in music performance. Similar findings emerged from Lucas et al.’s (2011) study of an Afro-Brazilian Congado performance in which the EMMP approach to methodology was also used. Despite active attempts made by members of two groups of musicians to resist it, entrainment was still found to occur on 50% of occasions when these groups came into close proximity of each other during a music performance. Group members were unaware that they became entrained with another group member and, again, visual contact played an important role in establishing entrainment, leading the authors to conclude that proximity is necessary but insufficient for entrainment.

That eye contact and gesture have been found to affect entrainment in music performance is no coincidence given the embodied nature of music performance (see Clayton and Leante, 2013). For Keller (2008, 2014; Keller et al., in press), basic entrainment underpins the more complex cognitive-motor skills that allow musicians to engage effectively in joint action (for more on joint action, see Sebanz et al., 2006; Sebanz and Knoblich, 2009). Through joint action, musicians anticipate, attend to and adapt to each other’s actions to control the dynamics of real-time interpersonal coordination. Keller (2008) posits that a music ensemble functions cohesively due to three joint action mechanisms anchored in entrainment: (1) anticipatory auditory imagery – a musician anticipates the sounds the he/she and her fellow musicians will produce (see Keller et al., 2006; Keller and Appel, 2010; Novembre et al., 2013); (2) prioritized integrative attention – a musician maintains awareness of the relationship between their parts and the parts played by others (see Keller, 2001); (3) adaptive timing – a musician consistently adjusts the timing of his/her sounds and movements to maintain synchrony with those of others. These three mechanisms then allow ensemble musicians to smoothly execute their shared performance goals – in instances where there are planned forms of co-ordination this may relate to their “idealized mental representations of the sounds constituting a musical piece” (Keller, 2014, p. 33) – in a way that works with the dynamic situational contingencies and restrictions they face in a particular performance.

Exploring the processes that underlie these joint action mechanisms, Phillips-Silver and Keller (2012, p. 3) differentiate between emergent and planned coordination, with the former entailing “spontaneous, automatic processes that are grounded in links between perception and action” and the latter requiring “shared representations of the intended outcome of the joint action” (ibid.) in addition to basic entrainment. The authors posit that the joint action behaviors of chorusing (separate individuals producing communicative signals that simultaneously and equally contribute to joint action) and turn-taking (when there is little temporal overlap between these communicative signals or when the signal produced by one individual is accorded priority over the signals produced by other individuals) in music are exemplars of emergent and planned coordination, respectively. The authors theorise that chorusing and turn-taking correspond to two different brain mechanisms, respectively: motor resonance and action simulation. Motor resonance is the bottom–up, automatic activation of movement-related brain areas triggered by the observation of the movement of another while action simulation is the “controlled, top–down activation of sensory and movement-related brain areas…in the absence of overt movement” (p. 3–4). In this way, entrainment is suggested to support joint musical action via two different routes, one that leads to emergent coordination such as chorusing via motor/perceptual resonance and the other that leads to planned coordination such as turn-taking via action simulation.

In sum, past research has established that entrainment plays a crucial role in music performance, with intra- and inter-group entrainment found to occur between performing musicians, even when attempts are made by these musicians to avoid them. Sharing strong ties with eye contact and gesture, entrainment underlies joint action mechanisms and their constitutive processes, all of which are responsible for musicians being able to perform smoothly together as an ensemble. While such findings provide solid evidence for the existence and importance of entrainment in music performance, the work of Doffman (2009, 2011, 2013) suggests that, while almost unavoidable, entrainment can also be actively used by musicians onstage as a performative device. Doffman (2009) used the EMMP methodology to study the relationship between entrainment and “groove” in the performance of jazz music. Defining groove as the “feeling of shared coherence and rhythmic flow that musicians look for in their playing together.” Doffman (2009, p. 131) posits that entrainment processes underlie the capacity for jazz musicians to groove together during a performance and that eye contact and gesture play their most important roles when groove begins to break down in music performance (see Doffman, 2011). Examining the presence of groove in the live jazz performance of a piano trio, Doffman (2013) conceptualises groove as an elastic equilibrium within an entrained relationship that, like entrainment itself, can recover from a disturbance. It is through groove, Doffman (2013, p. 83) suggests, that “musicians can explore and, to some extent, play with their mutual entrainment.”

Keil’s (1995; Keil and Feld, 1994) notion of participatory discrepancies – that there is an inherent messiness of creative tension at work within the fabric of music performance in any genre – forms the inspir ation for Doffman’s (2009) idea that musicians are able to play with entrainment through groove. Doffman (2009) describes how Keil’s (1995) captures in his idea of participatory discrepancies a tension that exists in ensemble music between each musician establishing an individual voice and portraying a sense of co-operation. This tension can be mitigated and used during performance by ensemble musicians if trust is established between them so that while the “fundamental cohesion of the group is at stake if the conscious manipulation of the time by one player is inappropriately used…in a situation where trust develops once players have worked with each other for long enough, the pushing or pulling of time becomes an important expressive/affective device…this sort of mild subversion of the cohesion has a paradoxical effect of increasing intimacy…here is an example of discrepancy as not simply the complex fabric of the music but an active distortion of that fabric to promote an increased sense of participation” (Doffman, 2009, p. 144). Provided trust levels are high enough amongst performing musicians, the suggestion here is that they can actively manipulate the disturbance-recovery mechanism on which entrainment hinges and use it during performance as a vehicle through which to convey an increased sense of expressivity, intimacy and participation.

Yet how would musicians’ use of entrainment processes as a performative device result in an increased sense of expressivity, intimacy and participation? When thinking through such questions, it is worthwhile considering the relationship between the audience and the performer/s. For Leman (2008), the audience and performer/s are also linked by entrainment processes. In his model of social music communication, he theorises that bidirectional sensory information channels run between the audience and the performer/s such that any actions made on stage influence listeners’ response to music, which then influences the actions produced on stage. Leman’s (2008) model can help explain Moelants et al.’s (2012) finding that performance becomes intensified when given in front of an audience. These authors compared data from one general rehearsal (no audience present) and one concert (audience present) recital of a performance given by a viola de gamba player and singer and found that although both performances were relatively similar, the performance in front of an audience featured exaggerated tempi (slower pieces were performed more slowly, faster pieces were performed more quickly) and an increase in the prevalence of open and communicative gestures adopted by the singer and in the overall intensity of the singer’s hand movements. In short, the audience’s presence led to the musicians generating a performance that was more performative than that which they gave in the audience’s absence, without being aware that they were doing so.

Drawing on Keil’s (1995) notion of participatory discrepancies, Doffman (2009) links the establishment of trust between jazz musicians with their ability to play with and use entrainment processes during performance. The improvisatory structure of jazz as a genre should not be underestimated as a factor at play in this relationship. Such a free-flowing structure might not only necessitate the establishment of high levels of trust between musicians but may also allow a space that enables musicians to *use* entrainment processes onstage as a performance device in a way that may be less possible within other genres of music. As we are looking at the possibility of similar processes occurring outside the genre of jazz, it is worth briefly examining the findings of Preston (2012) who explored improvisatory and collaborative practices during the songwriting process for rock musicians. Preston found improvisatory agents to organise their activity around three strategies during songwriting: appropriate-and-extend, proliferate-and-select and turn-taking. Musicians engaging in the appropriate-and-extend strategy creatively added to the sequence of actions that preceded the present moment in a way they viewed as being in keeping with situational expectations and constraints. Proliferate-and-select involves musicians extending the material they are provided with, generating a number of different options and then choosing between them during the songwriting process. Turn-taking^1^ can take place either at a macro-level via the explicit formulation of rules by a musician or set of musicians or at a micro-level where this formulation is much subtler and less conscious and can be determined by certain practices that have been established within the discipline or by something as pragmatic as the division of labor. Similar processes to those that guided Preston’s (2012) rock musicians during rehearsal may also have been operating for Doffman’s (2011, 2013) jazz musicians – both in any rehearsals they had leading up to a performance *and* during live performance – helping them to consolidate groove onstage and use entrainment processes performatively.

Past research that has investigated entrainment in music performance has tended to do so by analysing a case study of “real world” music performance under naturalistic conditions. Analysis of case studies of this kind has been established as an effective method through which to understand the “data-rich environments” (Doffman, 2011, p. 205) encountered within this field. Notwithstanding the prominence of case study analysis within extant research examining entrainment in music performance, Leman (2012) believes there is a need for even more case study research to be conducted within the area. Inspired by Leman (2012) and influenced particularly by the work of past researchers involved in the EMMP project, we adopted an ethnographic approach to studying entrainment in this project. The spirit of our research is in line with that of Lucas et al. (2011, p. 76) who describe how their research, “rather than a series of controlled experiments intended to test specific hypotheses... explores real-life data with both entrainment theory and ethnography in mind.” As our interest is in tracing the observable presence and effects of entrainment within a music ensemble, we chose to keep the analysis of our case study at the level of behavioral observation and interview data rather than combining this with detailed analysis of audio and visual performance data as the EMMP researchers did. The data comprising this case study were drawn from fieldwork that Geeves conducted as part of his doctoral thesis research that used Grounded Theory (Strauss and Corbin, 1998) to address the research question “What is the experience of music performance like for the professional musician?” (for more information on the methodology used in the thesis, please see Geeves et al., in press and Geeves, 2012).

The links shared by trust, entrainment and improvisatory practice in relation to live music performance by a music ensemble remain largely unexplored in past research. While previous research has engaged in rigorous cross-modal analysis of entrainment in specific sections of one particular music performance, we are not aware of any other published research that provides a diachronic profile of entrainment and of the development of trust within a group compiled from data that tracks the complete rehearsal and performance history of a single piece of music. We provide such a profile in our study, focusing on the way in which the last chorus of a song named “Stop” evolves from the time it is first learned and rehearsed by a musical quartet to the last time they perform it. In doing so, we examine the following research question: in what ways are trust, entrainment and improvisatory practice related to each other in live ensemble music performance? In the sections that follow, we introduce the subjects of our research and the procedure that we used to gather and analyse data. We contextualise and briefly profile the performance moment in the Results section and then track the way in which this section of music changed from its first rehearsal to last performance. Finally, in the Discussion section, we offer our account of the way in which entrainment, improvisatory strategies and the use of eye contact and gesture serve to establish a trust between the musicians in the case study that then allows them to use the inherent tension of entrainment during performance.

## MATERIALS AND METHOD

### PARTICIPANTS

**FIGURE 1:**
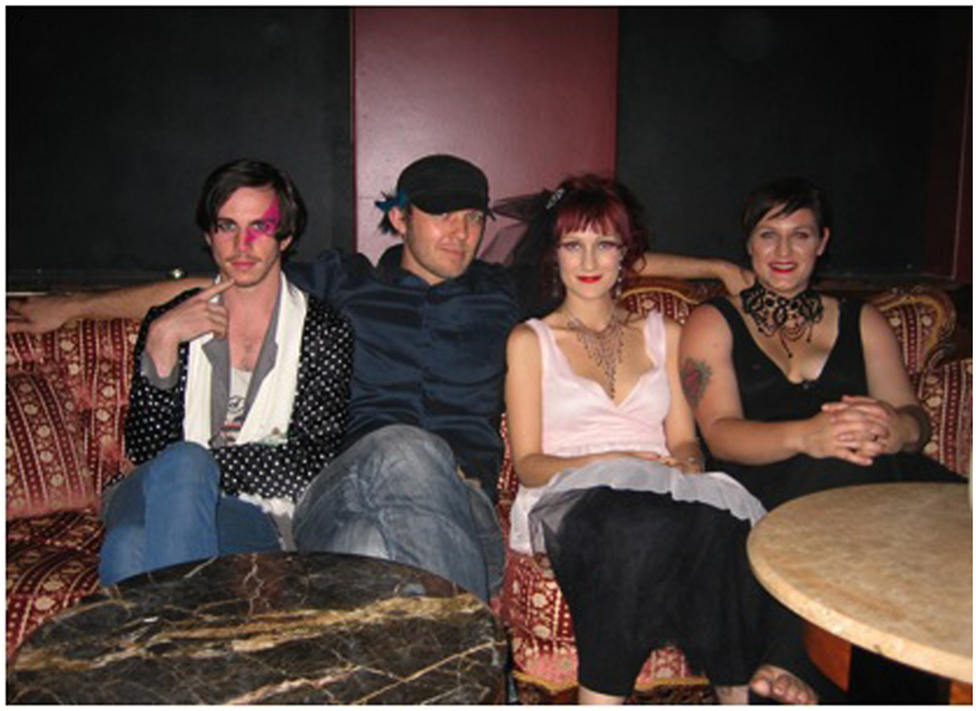
**WOFCT musicians (from left): Brendan, Ben, Emma, and Emily**.

Four professional^2^ Australian musicians consented to participate in the fieldwork conducted by Geeves (2012): Brendan Maclean^3^ (21, NSW), Ben Stewart (30, QLD), Emma Dean (25, QLD), and Emily Davis (27, SA; see **Figure 1**). Emma was the common link among the musicians as Brendan, Ben, and Emily had supported Emma when she performed in their home state during her previous national tours. Brendan, Ben, and Emily had not met each other prior to collaborating on the project on which this case study focuses. All four musicians traditionally performed solo, identifying their own style of music as fitting into a broad range of genres including indie, folk, alt country, pop cabaret, and blues. They hailed from a variety of performance backgrounds and possessed a diversity of beliefs about what music performance should achieve and reasons for why they performed music professionally.

### PROCEDURE

**FIGURE 2:**
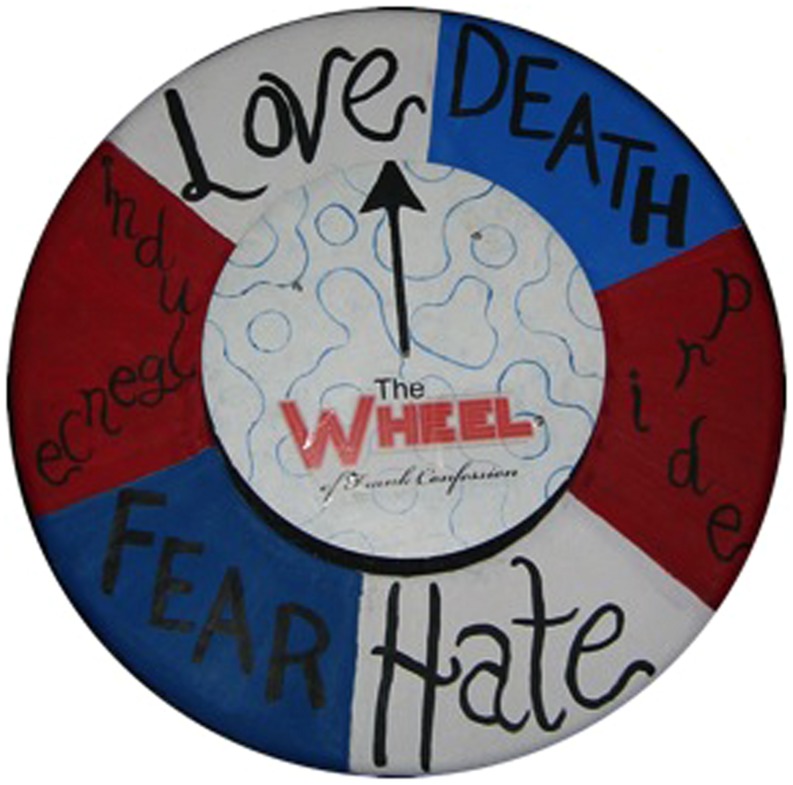
**The “Wheel of Frank Confession**.”

Ben, Emma, Emily, and Brendan performed as an ensemble in a collaborative project originally devised by Ben and Emma and named *The Wheel of Frank Confession Tour* (WOFCT). A hybrid of talent show and cabaret, the basic premise of WOFCT was that each concert would feature solo and group songs from the four performers. The solo songs performed on any given night would be dictated by audience members spinning a “Wheel of Frank Confession” that could land on one of six performer-devised “emotions”: love, death, pride, hate, fear, indulgence, or death (see **Figure 2**). A confession then had to be made to the audience by the musician whose turn it was to perform a solo song and this song had to correspond to the emotion spun up by the audience member. The group songs remained the same for every performance and anchored the show.

At no point in time did the musicians explicitly articulate to each other what they thought constituted a good music performance or what exactly it was that they were trying to achieve from their collaborative project (if anything). Although a loose overarching structure for the show was established before each performance, this structure changed between venues. The dynamics of each collaborative performance were even freer to vary and emerged from the combination of patterns that had been established via trial-and-error processes in rehearsals and prior performances with dynamic contextual contingencies that arose from performing to *this* audience in *this* space at *this* time. In this way, from the first rehearsal, there was an improvisatory feel to the WOFCT performance and, to a certain extent, the show was free to transform over the course of performances.

Geeves (2012) conducted fieldwork with the WOFCT musicians as they took their show on two separate tours of Australia: the first for approximately 2 weeks at the end of 2009 when the musicians traveled to Brisbane, Sydney and Melbourne (WOFCT1) and the second for ∼2 weeks at the beginning of 2010 (WOFCT2) when the musicians performed a series of seven performances at the Adelaide Fringe Festival. There was a period of approximately 3 months between WOFCT1 and WOFCT2. The WOFCT musicians had two rehearsal sessions together before each of their tours for which Geeves (2012) was also present. Geeves (2012) videotaped all WOFCT rehearsals and performances (yielding over 23 h of footage) and conducted semi-structured interviews with musicians throughout both tours (yielding over 9 hours of audio and video recordings). Geeves (2012) also made extensive field notes and diary entries (together totalling 35 338 words) for each day of WOFCT fieldwork, following Browne and Sullivan’s (1999) recommendation of maintaining detailed records to further establish credibility in qualitative research.

Geeves (2012) then transcribed and coded all semi-structured and fieldwork interviews following Grounded Theory guidelines (Glaser and Strauss, 1967; Strauss and Corbin, 1998). Geeves et al. (in press) coded each interview transcript separately to ensure reliability and validity in the coding and analysis process. They then met to compare their coding and discuss emergent themes. Geeves (2012) coded most transcripts by hand but some were coded using NVivo 8. The coding of the WOFCT interview transcripts was informed by the field notes and diary entries Geeves (2012) made. Sutton was privy to the entire coding and analysis process and provided valuable feedback and guidance throughout the project. As with other research that has taken as its focus the contents of subjects’ experience and used semi-structured interviews in its methodology (e.g., Downey, 2002, 2005, 2010 on capoeira and McIlwain and Sutton, 2014a on yoga), there is an inevitable discrepancy between that which a subject experiences and a subject’s report of that which he or she experiences. While data obtained from semi-structured interviews were analyzed in light of the field notes Geeves (2012) made while observing each musician perform, we are making inferences about the experience of performance from *reports* of this experience. Like any self-report data, these reports have the potential to be fallible and to exclude elements of a subject’s experience that are outside his or her conscious awareness (see McIlwain and Sutton, 2014b).

## RESULTS

The moment of performance at the center of this case study – the moment we describe in the Introduction – is the final chorus of the WOFCT song “Stop” from the ensemble’s fourth performance in Adelaide (henceforth referred to as “Adelaide Four”). “Stop” was a group song – a song that was planned always to be included in the show rather than being generated in response to an audience member spinning the “Wheel of Frank Confession” – that was led by Brendan and was the finale for the WOFCT2 performances. In this section, we trace how the structure of the last chorus of “Stop” evolved from the first time it was rehearsed, to the final time it was performed by the WOFCT musicians (see **Table 1**). This section can be read in conjunction with the links we provide to video footage of each moment that we mention (see **Table 1**) and contextualises our analysis of the case study in relation to entrainment and entrainment-related processes (found in the Discussion section).

**Table 1:** Evolution of the structure of the last chorus of “Stop” over the course of the WOFCT tour and accompanying video clips.

Performance	Date	Clip number	Number of repetitions by section of chorus line in last chorus of “Stop”		
			Brendan solo	Group breakdown	Group final
**WOFCT1**
Rehearsal 1	28/10/09	Clip one Clip two Clip three	4	2 and 4	4
Rehearsal 2	29/10/09		4	2	4
Brisbane 1	30/10/09		4	2	4
Queen St Mall	31/10/09		4		
Brisbane 2	31/10/09		4	4	4
Sydney	04/11/09		4	2	4
Melbourne 1	05/11/09	Clip four	4	2	4
Melbourne 2	06/11/09		n/a		n/a
Melbourne 3	07/11/09	Clip five	4	4	4
Melbourne 4	08/11/09		4	4	4
**WOFCT2**
Rehearsal 1	18/02/10	Clip six	4	2	4
Rehearsal 2	19/02/10		4	4	4
Adelaide 1	20/02/10	Clip seven	4	4	4
Adelaide 2	21/02/10		4	4	4
Adelaide 3	23/02/10	Clip eight	4	4	4
Adelaide 4	24/02/10	Clip nine	4	6	4
Adelaide 5	25/02/10		4	8	4
Adelaide 6	26/02/10		4	8	4
Adelaide 7	27/02/10	Clip ten	4	8	4

### THE STRUCTURE OF THE FINAL CHORUS OF “STOP”

The final chorus of “Stop” comprises a number of repetitions of the chorus line “Stop, pull in your head / the old, old world is dead.” The WOFCT musicians consolidated three distinct sections in the final chorus of “Stop” in rehearsals and performances leading up to Adelaide Four: (1) Brendan’s “solo” line repetition; (2) the “group” line repetition sung either a capella or with minimal piano and guitar accompaniment; (3) the “group” line repetition with fuller guitar and piano accompaniment. We label these sections: (1) Brendan solo; (2) Group Breakdown (because it is in this section that the group backs up Brendan’s soloing); (3) Group Final. Brendan solo and Group Final sat either side of Group Breakdown and were both rehearsed and performed at a set length of four repetitions of the chorus line. Up until Adelaide Four, Group Breakdown had been rehearsed and performed with a flexible length of either two or four repetitions of the chorus line (see **Table 1**)

### ”STOP” IN WOFCT1

Brendan taught “Stop” to the other WOFCT musicians in the first ever WOFCT rehearsal. Prior to this rehearsal, Brendan had sent a demo recording of “Stop” to Emma, Ben, and Emily so that they were familiar with the piece of music. Brendan had previously performed “Stop” in a live setting on a few occasions, sometimes performing solo and other times performing with a couple of accompanying musicians. At no stage did the WOFCT musicians explicitly discuss the previous performance history of “Stop.”

Clip one (http://youtu.be/5ttV8JoWIZs) is excerpted from the WOFCT musicians’ first ever rehearsal and captures the moment when Brendan begins teaching Emma, Emily, and Ben their “Stop” chorus parts. Ben is seen practicing his chorus chords in the background while Brendan teaches the melody of the chorus line, clarifies its lyrics and makes mention of a harmony to Emma and Emily. From this first rehearsal, the centrality of improvisation in the structuring of the last chorus of “Stop” is evident. “Is not there a harmony in there as well?” asks Emma after she and Emily have sung along to Brendan’s demonstration of the melody of the line they need to repeat in the chorus. Brendan replies, somewhat vaguely: “Yeah! Someone go up, someone go down....” Ben laughs and Emma smiles before asking, “So we can just muck around?” to which Brendan replies, “Rock out!.”

Clip two (http://youtu.be/DYmeRmpul9M) shows the musicians rehearsing the last chorus of “Stop” immediately after they had learned their parts in clip one. Brendan demonstrates a structure in which the chorus line is repeated ten times in the following way: (1) four “solo” repetitions of the chorus line by Brendan accompanied by claps on the off-beat from Emma and Emily (named “Brendan Solo from now on”); (2) two “group” repetitions of the chorus line in which Emma and Emily provide backing vocals and there is minimal instrumental accompaniment (named “Group Breakdown” from now on); (3) four “group” repetitions of the chorus line with backing vocals and now with fuller musical instrumental accompaniment (named “Group Final” from now on). In this clip, the musicians can be seen to “muck around” to learn and consolidate their parts. For example, at 1.13, Emma questions Brendan about a higher vocal harmony that she can sing to offset Emily’s lower vocal harmony and that she remembers hearing on the demo recording of “Stop.” Brendan sings this harmony a capella once for Emma and she replies “That’s it!.” Brendan then sings this higher harmony once more, adding in keyboard chords as accompaniment. Emma sings along with Brendan while Emily sings her lower harmony. The musicians also “muck around” with ideas about how the structure of the chorus might be open to change in future performances. From 2.24, Emily suggests that “Stop” would be appropriate to use as the WOFCT finale as it is “such a great jam-my song... (with) really great energy.” In a prescient moment, she also proposes that, in this finale, the musicians could “Do some blues-y vocals and **extend that last bit** (i.e., the “group” line repetitions) and go wild.” The other musicians agree with Emily’s proposal.

In clip three (http://youtu.be/YCiHHpLY8ww), the WOFCT musicians practice “Stop” in its entirety for a third and final time in their first rehearsal. Now that the musicians are more confident in their knowledge of the song, Brendan incorporates Emily’s suggestion of extending the first section of “group” line repetitions in the chorus. Brendan extends the Group Breakdown section by two more chorus line repetitions, making a total of eight “group” repetitions and 12 (rather than ten) repetitions of the chorus line in total. Notably, the WOFCT musicians consolidate the structure of the last chorus of “Stop” without ever explicitly articulating to each other in language that they are doing so. Brendan demonstrates Group Breakdown with two repetitions in the second practice of “Stop” and, following Emily’s suggestion, with four repetitions in the third practice of “Stop.” No part of the conversation between the four musicians refers to the mutual understanding they hold; that there is flexibility in the length of the Group Breakdown section or that there is no flexibility in the length of the other two sections. Although solid within the group, this mutual understanding is achieved only through the group’s practice of playing through “Stop” three times. The implicit agreements made between the WOFCT musicians in relation to “Stop” and its sections in this first rehearsal lay the foundations for future rehearsals and performances of this song. When the WOFCT musicians rehearsed together for the second and final time (for WOFCT1) the following day, a number of other songs were workshopped but “Stop” was practiced once only (with Group Breakdown lasting for two chorus line repetitions – see **Table 1**). After running through “Stop,” the group immediately moved on to playing another song. The lack of *any* discussion about “Stop” between WOFCT musicians during the second WOFCT1 rehearsal speaks not only to the marked difference between the two WOFCT1 rehearsals but to the confidence the WOFCT musicians must have had in the decisions they had reached about “Stop” through the series of implicit negotiations conducted in the first WOFCT1 rehearsal.

WOFCT1 performances of “Stop” featured a mixture of two and four repetitions of the Group Breakdown section in its last chorus (see **Table 1**). A sample of these performances is found in clip four (http://youtu.be/RtANGlEQ6DA) and clip five (http://youtu.be/0m17TatEB1U). These clips are excerpts from the last chorus of “Stop” from the first performance (clip four) and third performance (clip five) the musicians gave in the South Melbourne terrace that housed the now-defunct venue “The Butterfly Club.” With a capacity for 35 audience members, this venue was much smaller than the venues in which the WOFCT had performed in Brisbane (capacity 120) and Sydney (capacity 150). It is interesting to note that, in front of clip four’s small audience, Brendan opted only to repeat the Group Breakdown section twice. In contrast, the clip five audience was the largest and most responsive audience the performers attracted in Melbourne and, in this setting, Brendan chose to extend the Group Breakdown section to four repetitions. As in rehearsals for WOFCT1, during all WOFCT1 performances Emily, Emma, and Ben were comfortable following Brendan’s lead irrespective of whether he chose to double the length of the Group Breakdown section.

### “STOP” IN WOFCT2 REHEARSALS AND PERFORMANCES PRIOR TO ADELAIDE FOUR

In the two rehearsal sessions leading up to the WOFCT2 performances, both the two- and four-line versions of the Group Breakdown section were practiced (see **Table 1**). In these rehearsals, the musicians appear more comfortable playing together as a group and more easily able to anticipate, read and respond effectively to each other. This allows the musicians even greater freedom when “mucking around” with ideas for performance in rehearsal. Take, for example, the rehearsal moment captured in clip six (http://youtu.be/DYpFhPGuhqQ) at the end of the first WOFCT2 rehearsal. The group has just finished rehearsing “Stop” and Brendan is continuing to play through the chorus as Emily, off-screen, makes cups of tea for herself and the other three musicians. Ben is sitting against a wall with his guitar and Emma is “mucking around” with a ukulele she has found. As he reaches the Group Breakdown section, Brendan looks to Emma and says “Ukulele solo!.” The ukulele’s soft volume forces Brendan to lower the volume of his vocals and accompanying clicks for the first line repetition and similarly forces Ben, who has joined in by now, to strum his accompanying rhythmic guitar very softly. The pianissimo dynamic continues for Brendan’s next three line repetitions of the Group Breakdown section as Emma continues to play the ukulele and Ben and Emily experiment with singing higher backing vocals. Brendan then leads the group into a crescendo for the Group Final section in which he improvises the lyrics, rhythm and melody of a vocal line that lasts for four line repetitions and that has, until this rehearsal, seen little variation.

The venue for the Adelaide performances was an old, disused cinema complex adjacent to Rundle Mall that had been stripped of its furnishings. Although it had a capacity for the many hundreds of people held by a regular cinema, an area that was designed to hold a maximum of 60 audience members was cordoned off for Adelaide Fringe Festival performances. The number of audience members that the WOFCT drew to this space ranged from just over 30 (i.e., the venue was a little over half full) for their final performance to under 10 for some of the performances in the middle of their run that were scheduled at a time of 11 pm (much later than the performance time scheduled for WOFCT concerts at the beginning and end of the Adelaide Fringe Festival run). However, despite the WOFCT musicians performing in front of audiences that were diverse in both size and responsiveness, the two-line version of the Group Breakdown section never appeared during any of the Adelaide performances (see **Table 1**). Samples of these performances are found in clips seven and Eight (http://youtu.be/6rPvKo1wmX0 and http://youtu.be/T31s-Sx1vjE), taken from the first and third WOFCT performances in Adelaide, respectively, both of which feature four-line versions of the Group Breakdown section. In these clips, Brendan can be seen to be moving and altering the rhythm and melody of his vocal line more than he did in WOFCT1 performances. It is worth noting that the Adelaide stage had the biggest area of all the stages on which the WOFCT musicians performed and the greater range of movement available for Brendan to take on this stage may have influenced his actions.

### ADELAIDE FOUR PERFORMANCE

As seen in clip nine (http://youtu.be/zZXgCL2isv8), in the eleventh live performance of the WOFCT show Brendan breaks the conventions that the quartet had previously established to govern the structure of the last chorus of “Stop.” Without forewarning his fellow musicians, Brendan extends the Group Breakdown section by repeating the chorus line a further two times (six times in total). The other three musicians adjust to this change quickly and smoothly, following Brendan’s lead and allowing the performance to continue in a seamless manner.

### “STOP” IN WOFCT2 PERFORMANCES AFTER ADELAIDE FOUR

The WOFCT musicians’ ability to handle Brendan’s improvisation in the Adelaide Four performance set a precedent that allowed the musicians to continue to work together and follow Brendan’s lead in the last chorus of “Stop” in their remaining three Adelaide performances. However, despite never articulating the explicit aim of doing so, the group worked together over the final three performances on subsequent nights to expand the Group Breakdown section to *eight* repetitions of the line. This worked well for the fifth and sixth Adelaide performances however, in the seventh and last Adelaide performance, Brendan risked an improvisation that the group was unable to accommodate quite so smoothly. As seen in clip ten (http://youtu.be/JSXQbxB_b6Y), in the last chorus of “Stop” in the last WOFCT performance, Brendan risked improvising in a place that he had not improvised before: immediately before the last chorus begins. Before the first repetition of the chorus line at the beginning of the Brendan Solo section, Brendan gasps and dramatically slows the tempo of the song, bashing the keyboard to produce a discordant sound. This unanticipated action catches the other three musicians unaware as they were expecting to go straight into the chorus as they had in every other performance and rehearsal.

Each musician reacts in a different way to this unanticipated change. Ben hears Brendan’s gasp, looks over to him, notices that he is about to do something different, pauses and then tries – with a moderate amount of success – to time the strumming of his guitar to be in synch with Brendan’s erratic keyboard mashing. Emily clicks once on what would have been the off-beat had the tempo remained the same, realises something is amiss, looks over to Brendan and then swiftly bends her knees and bows her head, mirroring in her body the way in which Brendan is bringing a broken-down feel to the song. She then pauses and looks back over to Brendan to see what he will do next. Emma claps what would have been the off-beat, realises Brendan is doing something different, looks toward Brendan, adds a disjointed clap that mimics the jaggedness of Brendan’s keyboard playing and then becomes limp from the waist up, drops her head down over her feet and places her hands on her head. When Brendan strikes the first chord of the chorus, Emma raises her head, parts her hands to look over at Brendan and then drops her hands to her waist, rolling her eyes and shaking her head as she looks up. She then throws her hands up in the air in a wide “V” gesture of mock exclamation before bringing them together to clap on the offbeat while quickly glancing across at Emily and subtly shaking her head.

What occurs in the Adelaide Seven performance showcases the limits of “mucking around” that Brendan can engage in without throwing the other ensemble musicians and interrupting performance. Up until this point in time, there has been a cumulative expanding of the Group Breakdown section, with this process accelerating after the Adelaide Four performance. In his role leading the ensemble, Brendan has obtained the freedom to do almost anything he wants in the Group Breakdown section safe in the knowledge that his fellow musicians will be able to follow his lead smoothly. However, he obtains markedly different results in this performance when he makes an improvisatory attempt to exercise his freedom in *another* section of the chorus. In doing so, Brendan takes his fellow musicians by surprise and the performance threatens to unravel before the quartet manages quickly to regather and continue on without too much interruption to the performance. We now turn to an examination of the processes that allow the ensemble to regather quickly in the Adelaide Four performance and that allow them to follow Brendan’s lead so smoothly in other performances.

## DISCUSSION

Having established the trajectory of the evolution of the structure of the last chorus of “Stop” throughout the WOFCT tour, we now analyse processes that may have influenced this change. This case study exemplifies Clayton’s concept “intra-group entrainment” and, as in his example, it falls outside of conscious awareness for the participants. The entrainment processes emerge over time and on the fly, tacitly negotiated in gesture, rhythm of movement, spatial proximities and glance, but, in our case study, not explicitly discussed. Entrainment processes are only hidden in the sense that few of us are able to store and reflect on group performance processes over such a span of time and at such a detailed level. It is not so much unconscious in the sense of defended against and unknowable, but more not consciously tracked, and perhaps not able to be tracked without the reflective opportunities afforded by media. So our analysis of entrainment processes, rendering them explicit, is not a baring of the participants by the “expert” observers (which a concerned reviewer raised as a possibility). The data we use as the basis of our analysis are the product of rapport and intensive collaboration between Geeves (2012) and the performers. Our analysis explicitly tracks tacit negotiations between performers, using audio-visual media, notes and interview material to permit new reflective vantage points on complex, swift, high-density real world interactions occurring outside of language.

### AN OVERVIEW OF THE COMPONENTS OF ENTRAINMENT EXEMPLIFIED IN THIS CASE STUDY

First we use Phillips-Silver et al.’s (2010) framework to demonstrate the observable presence of entrainment and improvisatory practices in rehearsal and in performance. We then outline the spatiotemporal and contextual contributions to entrainment, and detail how it is entered into (in WOFCT rehearsals and performances), sustained and stretched via eye contact and gesture. We document how the way that entrainment processes change via smooth adaptation despite Brendan’s temporal stretching consolidates trust. We see this trust as a unique feature of entrainment in interpersonal settings. In line with existing research, we document how disruption is both part of and threatens entrainment processes permitting performers to play with the tension inherent in entrainment, in a performative manner onstage and in a way that at times includes audience members. Entrainment is asymmetrical in this case study, with the group leader being the major source of temporal stretching of the entrainment phases, disruption to the point of breaking the entrainment. Non-etheless, we document the establishment of a trust that allows musicians to play with the “tension between presence and absence” (Doffman, 2013, p. 64) that underlies entrainment in live music performance and, ultimately, to use it as an onstage performance device for increasing audience engagement.

### THE OBSERVABLE PRESENCE OF ENTRAINMENT: FOUR FORMS OF ENTRAINMENT IN THE LAST CHORUS OF “STOP”

Numerous examples of the presence of entrainment are apparent in the clips from WOFCT rehearsals and performances listed in **Table 1** and referred to in the Results section. These clips of observable instances of the four different types of entrainment identified by Phillips-Silver et al. (2010) are exemplified alongside a discussion of the way in which the turn-taking and chorusing behaviors described by Phillips-Silver and Keller (2012) are negotiated by the WOFCT musicians. Since Clayton’s (2012, 2013) intra- and inter-individual entrainment overlap entirely with the more detailed account offered by Phillips-Silver et al.’s (2010) four types of entrainment, we discuss only categories here. Clayton’s inter-group entrainment is not found in the WOFCT single group case study.

#### Entrainment in rehearsals

From the first WOFCT rehearsal, the presence of all of Phillips-Silver et al.’s (2010) four categories of entrainment is evident. In clip two, during repetition five of the chorus line (0.32), Brendan demonstrates *self-entrainment* when he begins to move his head back and forth in time with the beat that he is clapping. In response to this, Ben demonstrates *social entrainment* as he begins to move his head in time with Brendan’s head. Emma and Emily demonstrate *mutual social entrainment* as they sing the backing vocals together, their efforts to maintain entrainment particularly evident when Emma looks over to Emily so as to maintain the rhythm of their line as Brendan changes the rhythm of his line (0.46). *Collective social entrainment* occurs throughout the chorus via the clearly apparent interweaving of melody and harmonic, instrumental, vocal and rhythmic accompaniment. For example, at the beginning of the clip, Emma’s claps become entrained with Brendan’s melody line and keyboard (0.05) and these share a reciprocal relationship with Emily’s claps. As the chorus progresses, Emma and Emily’s backing vocals feed into Brendan’s lead vocals which feed back in to their backing vocals, all of which feeds into Ben’s guitar accompaniment which feeds back to all of the other musicians. Dramatically different contributions from each of the musicians give rise to the formation of a new, emergent whole.

In addition to learning how to play “Stop” together, the musicians can be seen to be delineating the way in which they will partake in the chorusing and turn-taking behavior described by Phillips-Silver and Keller (2012) in this first rehearsal. The entire last chorus of “Stop” features Emma, Emily, and Ben chorusing while Brendan turn-takes. Yet the amount of freedom that Brendan has to incorporate improvisation into his turn-taking behavior differs in each section (as outlined in the results, see The Structure of the Final Chorus of “Stop”section). By consolidating and reinforcing the three sections of the last chorus of “Stop” in their first rehearsal, the WOFCT musicians establish a protocol for how they will negotiate chorusing and turn-taking behaviors during performance.

#### Entrainment in the performance context

Phillips-Silver et al.’s (2010) four types of entrainment are clearly operative in the WOFCT performance context. In the performance captured in clip four, Brendan demonstrates *self-entrainment* by keeping his vocal and piano parts in time with each other and with the beat that he is marking by tapping his leg on the ground. *Social entrainment* can be seen when Ben continues to look over at Brendan’s head nodding and uses it as rhythmic input while Brendan looks down at the piano to concentrate on playing his own part (0.12). *Mutual social entrainment* occurs when Emma and Emily lock gazes and clap in synch (0.15) while *collective social entrainment* is found at 0.46 when Emma, Emily, and Ben adopt similar postures to each other that allow them to remain open to the audience and to face Brendan; all this while swaying in time to the beat of the music that Emma and Emily are highlighting by marking the off-beat on the tambourine (Emma) and by clapping (Emily). In this clip, musicians can also be seen to be following the protocol they established in rehearsals for their turn-taking and chorusing behavior.

Over the course of the WOFCT1 tour, the WOFCT musicians gained more experience playing as an ensemble, consolidating the ways in which they were becoming entrained together. There is greater freedom in their “mucking around” during rehearsals for WOFCT2. In clip six, the musicians demonstrate mutual social entrainment when they play around with instrumentation and dynamics in the chorus. Emma’s ukulele playing (from 0.08) feeds into Brendan’s lowering of his vocal volume to which Ben and Emily’s backing vocals adjust before all musicians adjust their dynamics to follow Brendan’s crescendo. Underpinned by the strengthening of entrainment processes, the expansion of the way in which the WOFCT musicians “muck around” to negotiate the chorusing and turn-taking behaviors during rehearsal shapes the last chorus of “Stop” in WOFCT2 performances. In these performances, Brendan takes greater liberties in his turn-taking behavior in the Group Breakdown section than he did during WOFCT1 performances.

### SPATIAL ATTRIBUTES OF ENTRAINMENT

Another advantage of studying a series of performances in different venues with varying audience sizes is that we can map the contribution of spatial features of context to entrainment. Brendan is seen in clip seven increasing the space that lies between him and his keyboard when he moves away from it in the Group Breakdown section as well as increasing his movements to the music. In clip eight, Brendan expands his movements again, crossing in front of his keyboard and the other performers for the first time in a performance, “lapping” the stage before returning to his keyboard. The strengthening of entrainment processes in the lead up to WOFCT 2 performances allows Brendan both to expand his movement onstage and to extend the Group Breakdown section out to four chorus line repetitions regardless of the size and responsiveness of the audience. Ultimately, this culminates in Brendan capitalising on this freedom to extend the Group Breakdown section out to six line repetitions in the Adelaide Four performance.

### THE ROLE OF GESTURE AND EYE CONTACT IN ESTABLISHING AND SUSTAINING ENTRAINMENT ILLUSTRATED VIA DISCUSSION OF THE LAST CHORUS OF “STOP”

In line with the findings of Clayton (2007b) and Moran (2010, 2013), use of gesture and eye contact was important during WOFCT performances and was most readily observable when entrainment was threatened by interruption. The centrality of gesture and eye contact to establishing and maintaining entrainment is established the first time the musicians run through “Stop” together. In clip two, Brendan nods his head on the first beat of the chorus (0.03), signaling to Emma and Emily two things: (1) this is where they should begin clapping the off-beat of the song and (2) the accompanying vocals they previously rehearsed briefly (in clip one) are imminent. Emma begins clapping while Brendan finishes singing the word “head.” Emma then begins clapping and asks Brendan – as he continues performing – “Should we be clapping?” before glancing across at Emily (0.05). Emily nods (off screen) and begins clapping, Emma nods back at Emily before Brendan looks at Emma and says “Yeah” (0.08). In return, Emma looks at Brendan and responds “Yeah” and then turns her head to make eye contact with Ben to ensure that he understands what is happening (0.09–0.10). Such a rapid-fire exchange of gestures and eye contact is typical between WOFCT musicians and can be observed in both the rehearsal and performance clips. However, the gestures the musicians make to each other are not always as obvious as Brendan’s head nod; there is a subtle lift of an elbow and curl of fingers in clip three, for example. Brendan begins to lengthen the Group Breakdown section for the first time by saying to the musicians “Another round, do another round” (0.40) at the end of the sixth repetition of the chorus line. As Brendan says this, Ben begins strumming the guitar rhythmically as he did in repetition six of the chorus line in clip two. Emma then looks at Ben and makes a subtle gesture whereby she lifts her right elbow and curls her right hand around to touch her right shoulder as she nods her head, indicating that Ben’s style of strumming is working in the song but that he should have entered with this strumming at the beginning, rather than the end, of the sixth repetition of the chorus line.

Eye contact and gesture play an especially important role in the WOFCT performance in the Adelaide Four performance, when Brendan’s unprecedented stretching of the Group Breakdown section threatens to disrupt the ensemble’s entrainment. Just before the sixth repetition of the chorus line, Brendan leaves his keyboard and walks behind the other three musicians (0.35). Emily glances to her right and notices Brendan walking behind Ben (0.38). As his fellow three musicians hold the note on which “dead” is enunciated, Brendan emerges between Ben and Emily. Looking toward Emma and Ben, Brendan makes a gesture in which he wiggles the extended fingers of both hands and lowers them from a level that is equal to his shoulders to a level that is just below them (0.40). Brendan uses this gesture – similar in its action to the gesture used to convey “rain” in the children’s song “Incy Wincy Spider” – to capture the attention of the other musicians and convey to them that he wants the group to decrescendo in (i.e., reduce) their volume. Ben immediately notices this gesture and turns his head to the left to acknowledge this by making eye contact with Brendan (0.41). Continuing to clap the off-beat, Emily slightly turns her head toward Brendan, registering his gesture and its meaning (0.42). Emma, continuing to enthusiastically mark the off-beat on tambourine, has not yet seen Brendan’s gesture. Brendan looks over at Emma, his hands again raised to be level with his shoulders (0.42). Continuing to look at Emma in a bid to capture her attention, Brendan continues to wiggle his fingers, closes his palms on the off-beat on which “Stop” is enunciated, clicks the next off-beat and then, as the words “pull in your head” are sung, makes the same gesture he did at the end of the sixth repetition. Emma notices this gesture, makes eye contact with Brendan and markedly reduces the volume of her backing vocals (0.44).

Now that the group has successfully executed a decrescendo, Brendan clasps his fists together tightly underneath his chin, raises his eyebrows and shoulders and makes tip-toe movements, possibly to highlight to the audience and his fellow musicians the softness of the current dynamics (0.46). The chorus line is then repeated for an eighth time as Brendan moves forward to begin his improvised, newly extended solo section that will continue for the total duration of two line repetitions. On the ninth repetition of the chorus line, Brendan repeats the words “It’s dead” as, bending down slightly, he marks the off-beat by making a gesture with his left arm and hand that looks similar to the type of movement that is used to start a pull-chord lawnmower motor (0.55). As Emma, Ben, and Emily continue their quiet accompaniment, Brendan continues his solo at full volume. As Emma, Ben and Emily sing “dead,” Brendan turns his palms up and, as he turns to run toward the keyboard, begins moving them between being held out at a right angle to his waist to almost touching his shoulders (1.01). This gesture signals to the other musicians that it is time for them to crescendo as the a capella Group Breakdown section is coming to an end.

We suggest that such density of information is conveyed between musicians for these 40 s (0.41–1.21) in clip eight because it is during this time that there was the biggest threat to a disruption in ensemble entrainment. When Brendan leads the group into a decrescendo, within only 4 s he has conveyed a large amount of information to the other musicians about the new structure of the piece through gestures that Clayton (2007a) would classify as “Markers.” The musicians, in turn, have communicated to him that they have received this information. Through such concerted and rapid use of eye contact and gesture, Brendan ensures that the musicians are paying visual attention to him. Just as eye contact facilitated entrainment for Clayton’s (2007b) tanpura soloist and Lucas et al.’s (2011) Congado musicians (even when they were actively resisting this), so too do Brendan’s efforts at a gestural level to ensure that his fellow musicians are actively watching him help to guard against the threat of a break in their entrainment.

### TEMPORAL STRETCHING AND THREATED DISRUPTION OF ENTRAINMENT PROCESSES

The break in entrainment threatened during the Adelaide Four performance is realized in the section of the Adelaide Seven performance found in clip ten. The strength of the entrainment that has built up amongst the ensemble over the course of WOFCT rehearsals and performances has allowed Brendan the freedom to do almost anything he wants in the Group Breakdown section without risking interruption to the performance. Brendan knows from experience that his fellow musicians will be able to follow him and they expect to follow whatever it is that Brendan chooses to do. Yet, when Brendan tries exercising a similar flexibility in his turn-taking behavior in the lead in to the “Stop” chorus, it does not work anywhere near as smoothly. By attempting to exercise a level of freedom in a section where such freedom has not been present in previous rehearsals or performances, Brendan takes his fellow musicians by surprise. As Brendan acts outside protocol that the WOFCT musicians established around how he is expected to turn-take and how they will chorus, there is the threat of a major break in the music and of the performance unraveling. It is on account of the quartet being entrained that they manage to recover quickly. However, the Adelaide Seven performance showcases the limits of the flexibility that the WOFCT musicians have established to afford Brendan in his turn-taking and demonstrates what it looks like for entrainment to be disrupted (and then regained) amongst WOFCT musicians during performance.

As Clayton (2012) emphasises, entrainment hinges on disturbance, rather than a static precision of systems being in-phase. Systems need to be able to move out of phase and stabilise back into a phase relationship. What we see here is the way that in human systems this negotiation can be conducted at a non-verbal level involving facial expressions and bodily gestures which smooth the transitions. Playing with the tension of entrainment is a form of restrained freedom akin to improvisatory practice.

### IMPROVISATORY PRACTICES IN THE LAST CHORUS OF “STOP”

Improvisatory practices hinge on unexpected contingencies, “performative discrepancies” (Keil, 1995) that are played with as part of the risk of disruption of stable phase relationships. These improvisatory practices are clearly evident throughout the WOFCT rehearsals in relation to the last chorus of “Stop.” The only time in rehearsal that Brendan pauses the song and explicitly teaches his fellow musicians their parts is captured in clip one. The rest of the teaching and learning for “Stop” takes place within the context and momentum of the song being played through. In rehearsal, the musicians “muck around” with melody, harmonic and rhythmic accompaniment and structure. Together, the musicians set parameters for how “Stop” will be performed, confirming to each other either in the song or via a few words after the song has finished whether they will keep or discard the various ideas that they are trying out in rehearsal. Via this playful process of trial-and-error the musicians exercise their creativity, work collectively to generate a truly communal product and are able to reach consensus about how to encode the material they are learning and how to retrieve it in future performances.

By learning and rehearsing “Stop” by “mucking around,” the WOFCT musicians are negotiating, agreeing upon and consolidating shared standards about how they will perform the piece in ways that align with Preston’s (2012) three improvisatory strategies. In clip two, for example, at 1.13, Emma engages in the appropriate-and-extend strategy when she discusses with Brendan her vocal harmony part. In turn, Brendan employs Preston’s (2012) turn-taking strategy when he replies “And I’ll probably be doing something like this” as he demonstrates the piano part he will be playing for the first “group” repetition of the line. The beginning of a proliferate-and-select strategy can also be observed in this clip as the musicians “muck around” with ideas about how the structure of the chorus might be open to change in future performances. The musicians continue to engage in a proliferate-and-select strategy in clip three as they negotiate the conditions around Brendan improvising in the last chorus of “Stop.” Through this process, the musicians establish implicit rules about which various musical elements should be coupled and/or de-coupled, when this should occur in “Stop” and the extent to which this process is flexible and open to variability in various sections of the last chorus of “Stop.” These limits, non-verbally negotiated, form the basis of entrainment phase shifts.

### TRUST AS A BASIS FOR ENTRAINMENT: THE ESTABLISHMENT OF TRUST AMONGST WOFCT MEMBERS

For the WOFCT musicians, the processes that are occurring around the final chorus of “Stop” lie mostly outside the realm of language and explicit verbal discussion. For the most part, entrainment, improvisatory strategies and use of eye contact and gesture occurred implicitly within music rehearsal and performance. Despite being pressed on it, none of the musicians in any of the interviews conducted by Geeves (2012) articulated any kind of detailed planning behind or understanding of the evolving happenings during the last chorus of “Stop.” The only time the musicians discussed what occurred on stage during the Adelaide Four performance was when they were asked about it immediately after the performance. Below is an excerpt from this interaction:

Geeves: How did you guys know what to do when Brendan [extended the section]…?

Ben: I think everybody knows that when a chord gets quieter you drop down with it, you follow the dynamics of it.

Emily: I’ve never had you [Brendan] stand that close to me either, so I knew that something was going on.

Geeves: And how did you know that they were going to follow you, Brendan?

Brendan: Just trust.

Emma: And if it does not happen, you have to think of something else.

Brendan: Yeah, I would’ve thought of something else.

The knowledge that the musicians possess in relation to the Adelaide Four performance is related to the trust that Brendan describes. Ben refers to a shared knowledge about dynamics amongst the musicians that has been established on the WOFCT tour through moments such as that captured in clip six. Emily’s knowledge that something different is about to happen in Adelaide Four is based on her understanding – built from cumulative experience – of Brendan’s regular positioning on stage. Emma’s expectation that something else would be able to be thought of if the musicians didnot follow Brendan and Brendan’s confidence that he would have been able to think of something else also speaks to the sense of interconnectedness that has been built amongst the WOFCT musicians over the course of their rehearsals and performances. This increasing sense of cohesiveness amongst the ensemble members can be observed as the tour progresses. The musicians experiment more with “mucking around” and Brendan takes greater risks with his turn-taking in WOFCT2 rehearsals than in those for WOFCT1. The taking of such freedoms is indicative of the trust established amongst the musicians over the course of the WOFCT tour. Grounded in the entrainment processes consolidated between WOFCT musicians, this trust was also facilitated by the improvisatory nature of their performances which forced musicians to rely on each other in ways that would not have been necessary if a tighter musical structure was in place. This trust continued to be consolidated by musicians’ use of eye contact and gesture to communicate important performance information to each other while they were on stage.

A crucial feature of the trust that was established between the WOFCT musicians was their acceptance of the asymmetry of entrainment (see Clayton et al., 2005) brought about by Brendan’s adoption of a leadership role. Just as the soloing role of the guitarist in Doffman’s (2009) jazz trio meant that she was the trio member who brought tension to the performance by actively pushing the boundaries of time, so too did Brendan’s role as ensemble leader in “Stop” mean that he had greater influence than the other musicians in shaping its last chorus and playing with inherent musical tensions (see Keil and Feld, 1994; Keil, 1995). The musicians manage this asymmetry of entrainment by establishing the protocol around Brendan’s turn-taking and Emma, Emily, and Ben’s chorusing in rehearsals in a way that is analogous to Tribble’s (2011) notion of fluent forgetting. Just as a professional Shakespearian actor substituted words within the rhythmic framework of a play when he forgot a line by demonstrating a “fluid ability to adapt and shift within a highly constrained structure” (Tribble’s 2011, p. 76), so too do the musicians, within their negotiated structure for turn-taking and chorusing, have to meld to Brendan’s whim in the Group Breakdown section. In this section, Emma, Emily, and Ben trust that Brendan will lead them and, in turn, Brendan trusts that they will follow him. This trust underpins Emma, Emily, and Ben’s smooth adaptation around Brendan’s improvisation in the Adelaide Four performance. They follow Brendan’s lead wherever it goes because they are expecting (and he is expecting them) to do so. They trust that he will not lead them astray and he trusts that they will be able to adjust to accompanying him wherever he goes. Similarly, Brendan’s breaking of this trust results in the momentary disruption of the Adelaide Seven performance. By attempting to turn-take in a different section of the chorus and in a way that is outside even the loosely established protocol for such behavior, Brendan makes it impossible for the other musicians to accommodate his improvisation. The entrainment between the ensemble members is temporarily broken, but the gestures enacted by the chorus members where they play broken puppets, and non-verbally exclaim at the break in entrainment incorporates the rupture in a performative way, to some degree.

The way that disruption is handled in this case study exemplifies the ways in which entrainment does not remain at the level of the content and timing of music, but incorporates the embodiment of the performer in terms of eye contact, gestures, dynamic spatial relations (and the meaning of changes in those relations). Further, it extends to an experience of trust that permits greater freedom and play.

### PLEASURABLE IMPRECISION: MUSICIANS’ PERFORMATIVE USE OF THE TENSION INHERENT IN ENTRAINMENT IN THE LAST CHORUS OF “STOP”

We have tracked how the presence of entrainment processes, improvisatory strategies, and use of eye contact and gesture in WOFCT rehearsals and performances established trust amongst Brendan, Emma, Emily, and Ben. We now posit that this trust is what allows the WOFCT musicians to use the inherent tension underpinning entrainment in music performance, as a performative device that aims to increase audience engagement with a performance. By establishing protocol for chorusing and turn-taking behavior in rehearsals, the WOFCT musicians construct a set of rules that allows them to manage the risk of the breaking of entrainment processes during performance while also ensuring that they craft this tension into their show in a way that will be entertaining for the audience. Like the will-he-or-would not-he thrill that an audience experiences when watching a tightrope walker or lion tamer, a pleasurable tension is caused for an audience by a visible threat to entrainment processes between musicians during performance because this also threatens to disrupt the entrainment relationship the audience shares with the performers. Emily described the reciprocal nature of the relationship shared between performing musicians and their audience, echoing Leman’s (2008) notion of the entrainment relationship shared between the two parties:

The audience is so responsive. They give you a chunk of stuff and that lets you construct your song with that energy. So you give it back twice and then they build and build and build and build. We’re all building together. It’s like a convection current of energy.

On account of the inextricable links built between audience members and performing musicians, the trust that was established between WOFCT musicians allows them to use the constitutive tension of entrainment as a performance device. Just as the two musicians in Moelants et al.’s (2012) study amplified elements of their performance in front of a live audience, so too does the trust between WOFCT musicians allow them to bring to the fore the tension underlying entrainment and use it to enhance the performativity of their performance.

Such tension is palpable in the Group Breakdown section of the last chorus of “Stop,” particularly in performances like Adelaide Four. The audience is not privy to the knowledge that the WOFCT musicians have of the plan that they have established for Brendan’s improvisation in this section. At an implicit level, audience members entrained with the performers will be feeling the tension entailed by the looming threat of a break in entrainment; will the musicians – not to mention the audience – recover from this perturbation? Are these systems truly entrained? The musicians capitalise on this tension, using the threat of a break in entrainment to draw the attention of audience members in to their performance and, hopefully, to keep it there. The consolidation of trust amongst WOFCT members leads to greater confidence and ability in executing this performance strategy. In WOFCT1 performances, Brendan only lengthens the Group Breakdown section in front of larger and more responsive audiences. It is as though the strength of interconnectedness between the WOFCT musicians at this stage is still significantly dependent on the energy of the “chunk of stuff” that they are receiving from the audience. However, as trust increases amongst the WOFCT musicians, they become increasingly able to play with this tension regardless of the input they are receiving from the audience. Regardless of the size and responsiveness of the audience, the Group Breakdown section was extended for all WOFCT2 performances.

This illustrates the role of what Keil and Feld (1994; Keil, 1995) terms “participatory discrepancies” in establishing the limits of trust based on an ensemble’s acquired capacities to move out of an established phase relationship and into a new one. This ensemble illustrates the way that skill in improvisation in a performative context requires integrative attention (Keller, 2001), where a musician maintains awareness of the parts they play with the parts played by others, as well as adaptive timing.

## CONCLUSION

Entrainment requires systems to have some form of shared oscillatory activity, for them to be coupled in some way. This case study demonstrates the role that intra-group non-verbal communication plays in establishing and sustaining entrainment. Yet, since entrainment hinges on the notion of disturbance, as Clayton (2012) emphasizes, there is not the requirement of systems falling precisely into phase with each other, but rather a dynamic stabilization of a phase relationship. It is precisely this feature of entrainment that is exemplified in the tracing of entrainment across a series of rehearsals and performances of a single chorus. Following Doffman’s (2011) suggestion, we have offered a fine-grained analysis of real world performances. These performances occurred over time and in different venues, permitting us to address the spatiotemporal features of entrainment, which Leman (2012) suggests must be included for a comprehensive understanding of the phenomenon in music performance.

In our diachronic case study we have examined how the trust arising among WOFCT musicians born of entrainment, improvisatory strategies and use of eye contact and body gesture permits these performers to use this tension in a performative way that extends beyond performers to audience. Crucially, the mechanisms underpinning this trust are found to be largely indirect rather than explicit, involving more embodied processes of alignment and interpersonal coordination than direct instruction and decision. Rhythmic grooves of co-aligned independent elements in different musical registers are both planned and emergent to varying degrees, negotiated non-verbally on the fly (by glance, gesture, marked shift in tempo or volume) and received as part of the performance by changes in performance, by bodily synchrony of posture, or by attunement to the parameters of entrainment in voice or instrument. The dynamic shape of entrainment, and the elements that make it up, shift over performances and within a single performance. The threatened rupture and smooth adaptation become part of the performative medium. It is what Doffman (2009, p. 144) terms a “mild subversion” of cohesion, an “active distortion” of the fabric of music “to create an increased sense of participation.” Both arising from trust and sustaining it, entrainment is highly reliant on non-verbal negotiation and underpins the “live” quality of music performance.

## Conflict of Interest Statement

The authors declare that the research was conducted in the absence of any commercial or financial relationships that could be construed as a potential conflict of interest.

## Footnotes

1 It is important here to emphasise that Preston’s (2012) definition of “turn-taking” is very different from Phillips-Silver and Keller’s (2012) understanding of the term.

2 Given the contention that surrounds the process of defining who should be considered a “professional musician,” no widely accepted definition of this term currently exists. In this study, all participants were considered to be professional musicians because members of the public were willing to part with the price of admission to watch their performances.

3 Approval from Macquarie University Ethics Committee was obtained before each stage of research in this study was carried out and each musician also consented to being identified and directly quoted in future publications.
